# A novel phased approach to rapid response capacity strengthening in public health emergencies: Egypt’s experience

**DOI:** 10.1136/bmjgh-2025-019008

**Published:** 2026-01-08

**Authors:** Sherif Shamseldein, Mays Shamout, Baher Eldesouki, Sarah Malik, Asma Essa, Sherein Elnossery, Amr Kandeel

**Affiliations:** 1 Preventive Medicine and Public Health Sector, Egypt Ministry of Health and Population, Cairo, Cairo Governorate, Egypt; 2 Center for Global Health, Centers for Disease Control and Prevention, Atlanta, Georgia, USA; 3 Centers for Disease Control and Prevention, Atlanta, GA, USA; 4 PH Emergency Management Center, Eastern Mediterranean Public Health Network, Amman, Jordan; 5 Infectious Hazard Prevention and Preparedness Unit, World Health Organisation Regional Office for the Eastern Mediterranean, Cairo, Cairo Governorate, Egypt; 6 Deputy Minister of Health and Population, Egypt Ministry of Health and Population, Cairo, Cairo Governorate, Egypt

**Keywords:** Global Health, Health systems evaluation, Public Health, Infections, diseases, disorders, injuries, Epidemiology

## Abstract

**Introduction:**

Egypt’s Ministry of Health and Population adopted the US Centers for Disease Control and Prevention’s Analyze, Design, Develop, Implement, and Evaluate (ADDIE)-phased approach to strengthen and institutionalise rapid response teams (RRTs), moving beyond ad-hoc training toward sustainable systems. Using the Capacity Assessment Tool (CAT), Egypt identified operational and programmatic gaps, designed tailored workplans and implemented structured activities to enhance RRT readiness. This systematic process improved coordination, sustainability and capacity building, aligning with International Health Regulations and strengthening Egypt’s overall preparedness and response to public health emergencies.

**Methods:**

This longitudinal study measured RRT programme capacity and operational levels in Egypt from March 2022 to May 2023. Egypt RRT focal point and newly appointed managers used the CAT to evaluate 60 indicators across eight programmatic areas. The study used a before-and-after analysis of the three-phased interventions: RRT management training and Standard Operating Procedure development (July 2022), staffing and training of RRT (August 2022) and monitoring and evaluation (M&E) frameworks development (May 2023). Capacity and operational level scores measured across areas with scores ranging from 0 to 2 for each indicator.

**Results:**

Between baseline and the end of the intervention, the most significant improvements occurred in M&E (mean increase 1.80; p=0.001) and postdeployment (mean increase 1.50; p=0.007) capacity levels. For operational levels, statistically significant increases were observed across most areas, with the greatest improvements in postdeployment (1.33; p=0.01), activation and predeployment (1.27; p<0.001) and deployment (1.12; p=0.001). Results revealed that 41.7% of indicators showed two-point improvements in capacity, while 25% showed two-point improvements in operational levels. No indicators showed a decline in either capacity or operational performance.

**Conclusion:**

The structured ADDIE-based phased approach demonstrated effectiveness in enhancing both capacity and operational levels of Egypt’s RRT programme. Challenges persist in administrative considerations, highlighting the importance of addressing policy development and sustainable funding early in capacity building efforts. This approach offers a replicable framework for countries seeking to strengthen emergency response capabilities.

**WHAT IS ALREADY KNOWN ON THIS TOPIC**

- Public health emergency preparedness systems faced a significant gap between assessed capacity and actual operational performance during crises, as seen when strong Joint External Evaluation scores failed to predict effective COVID-19 responses.
- In Egypt, RRTs were first introduced during the 2005 avian influenza epidemic. However, these initiatives largely emphasised individual skills rather than building sustainable institutional capacity.

**WHAT THIS STUDY ADDS**

- This study demonstrates the effectiveness of a novel approach to rapid response capacity using the Analyze, Design, Develop, Implement, and Evaluate model.
- It provides quantitative evidence of improvements in capacity and operational levels across multiple programme areas and identifies optimal timing for different types of improvements.

**HOW THIS STUDY MIGHT AFFECT RESEARCH, PRACTICE OR POLICY**

- Findings suggest shifting emergency response capacity building from individual training to systemic institutional development while addressing administrative factors and sustainable funding early in programme design.
- This research offers a blueprint for sustainable RRT programmes in national health systems.

## Introduction

 Public health emergency (PHE) preparedness and response is a multisectoral effort requiring strategic public and private partnerships encompassing global, regional, national and local cross-functional collaboration.^1^ In 2016, the Joint External Evaluation (JEE) was presented to assess a country’s capacity to prevent, detect and respond to public health emergencies.^2^ Global COVID-19 response performance highlighted how preparedness and response readiness in JEE do not imply coordination and operational performance during a public health response.^3^ This incongruency between evaluated emergency preparedness and response capacity and operational performance highlighted a gap and critical need for a standardised approach to sustainable and operational capacity building. To fill this gap, the US Centers for Disease Control and Prevention (CDC), in 2021, developed a systematic phased approach to emergency response capacity building adopted from an instructional design framework, the Analyze, Design, Develop, Implement, and Evaluate (ADDIE) model.^4^ The ADDIE model is a flexible, systematic and cyclical process traditionally used for designing training. Since capacity development is an ongoing process, the CDC sought to implement the ADDIE model in their capacity building work, ensuring a standardised but tailored approach to capacity building in the supported countries, allowing for the ability to measure impact.^5^

Public health rapid response teams (RRTs) are multidisciplinary teams trained and equipped to rapidly deploy to a PHE in coordination within a larger emergency response framework.^6^ Using an interdisciplinary approach, RRTs can be an asset within a country’s PHE response system and can be used throughout all stages of an outbreak response. During the avian influenza epidemic (H5N1) in Egypt, the Ministry of Health and Population (MoHP) emphasised the endorsement and activation of the multilevel RRT network in its 2007 national avian influenza preparedness plan to ensure that PHE response systems were in place to manage the response activities.^7^ Following the influenza epidemic, Egypt’s rapid response efforts revealed several gaps, such as limited surge capacity for rapid deployment, inadequate coordination between national and subnational levels, insufficient standardised operational procedures and challenges in maintaining trained personnel rosters. Postoutbreak evaluations identified weaknesses in multisectoral coordination, particularly between human and animal health sectors and highlighted the need for sustained funding mechanisms to maintain response readiness. These experiences underscored the limitations of ad hoc training approaches and the necessity for systematic institutional capacity building. Since the avian influenza epidemic, Egypt has responded to numerous public health emergencies and has conducted various RRT training to build preparedness and response capacities at the national and subnational levels.

Multiple public health outbreaks in Egypt highlighted gaps in preparedness and response. A large salmonellosis food poisoning event linked to poor hygiene practices affected hundreds of students, while an active malaria focus emerged due to imported cases, requiring intensive surveillance and rapid treatment to prevent reintroduction. Dengue virus reappeared after decades, but swift vector control contained its spread to a few villages. A measles outbreak among undervaccinated nomadic children underscored the risks of low immunisation coverage, prompting mass vaccination campaigns. Additionally, a large-scale botulism outbreak associated with traditional Egyptian dish and the response to the COVID-19 pandemic.^8 9^

In all these situations, resource and capacity limitations were consistently identified across outbreaks. There were insufficient resources in terms of expertise, drugs, vaccines, laboratory services, equipment and other facilities necessary for effective outbreak management. The absence of uniform standard operating procedures for RRT activation in outbreak management created delays and inefficiencies. Additionally, there was a lack of officially formed national RRT with clearly specified roles and responsibilities for members.

These experiences revealed the critical need for shifting from focusing on individual and team capacity building to institutionalising comprehensive response systems. This shift is imperative, as it ensures readiness is not a function of individual expertise but integrated within the public health infrastructure through developing a sustainable RRT programme.

The PHE response structure in Egypt operates through a multitiered system coordinated by the MoHP’s Preventive Medicine and Public Health Sector. At the national level, the RRT Unit serves as the central coordination hub, working closely with the national Public Health Emergency Operations Center (PHEOC). Subnational response is organised through governorate-level health directorates, which coordinate with district and local health units. During public health emergencies, this system activates RRTs that can be deployed from national, governorate or district levels depending on the nature and scope of the emergency. The RRT programme operates within this existing framework, enhancing coordination capabilities and ensuring standardised response procedures across all levels.

In addressing this priority, following systemic methodology with a defined framework for systematic enhancement was imperative. For this, Egypt adopted the CDC’s structured ADDIE approach to establish an institutionalised RRT programme within the country’s MoHP’s Preventive Medicine and Public Health sector. In March 2022, Egypt MoHP used the CDC Capacity Assessment Tool (CAT) to assess their RRT programme’s capacity and operational function as part of the first phase of the approach, the analysis phase. The CAT is a comprehensive, single document designed to assess a country’s readiness for public health events by identifying opportunities to address gaps in the emergency preparedness and response system’s capacity.^10^ While the JEE assesses a country’s capacity to implement International Health Regulations (IHRs) by evaluating the existence of systems and structures at the national and subnational levels, it lacks depth in assessing how effectively they work in practice during public health events. Egypt’s use of CDC’s CAT allows for a deeper dive into programme level capacities and operational level that helps identify practical gaps and guides a tailored, actionable workplan with the ADDIE approach, which aims to build sustainable capacity and its implementation. The CAT assesses a country’s RRT programme through indicators outlining the establishment and management of RRT-specific plans, policies and procedures, RRT programme recruitment, onboarding, training, predeployment, deployment and postdeployment processes and One Health considerations are also included. Following the assessment, a work plan was devised using the CDC’s phased approach to capacity building to assist in establishing and developing their RRT programme capacity with technical assistance from external partners such as the CDC, Global Health Development, WHO Egypt country office and WHO Eastern Mediterranean Regional Office.^11^

**Figure 1:**
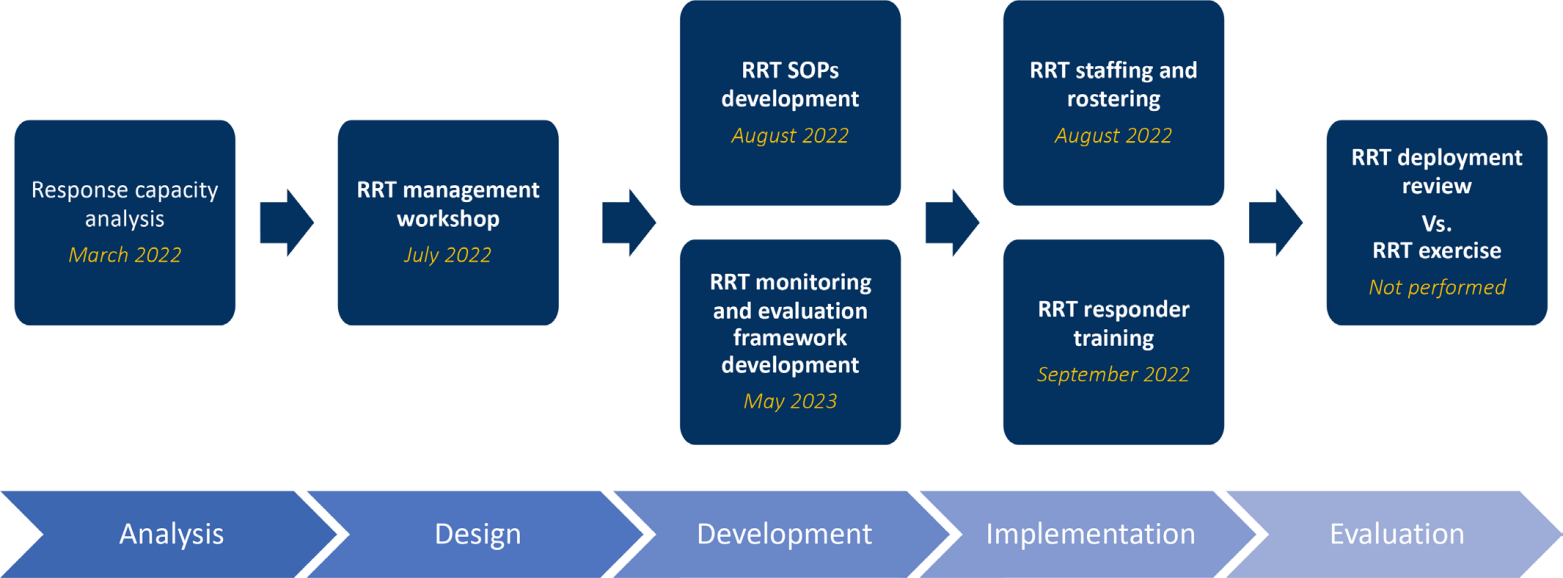
Egypt’s phased approach to their Rapid Response Team Program capacity building following the ADDIE model. ADDIE, Analyze, Design, Develop, Implement, and Evaluate; RRT, rapid response team; SOP, Standard Operating Procedure.

This phased approach provided Egypt with a roadmap to methodically analyse, operationalise and integrate existing emergency response resources into their national RRT programme capacity building (figure 1). This technique for emergency response capacity building complements IHR and JEE indicators, strengthening a country’s ability to effectively prepare for, respond to and recover from public health emergencies and meet global benchmarks.^1 2^ To identify the capacity-building impact of the phased approach, the Egypt RRT programme conducted an additional capacity assessment after each of the three above-mentioned activities using the CDC’s CAT. This paper answers questions regarding capacity improvement and stability in the capacity and operational level of the Egypt RRT Programme after the three capacity building activities. This paper will also highlight which capacity-building activities provided the most improvement in capacity and operational level.

## Methods

### Capacity assessment tool

In February 2022, an orientation to the CDC CAT was provided to Egypt MoHP leadership, specifically targeting the newly established RRT Unit in the Preventive Medicine and Public Health Sector, and senior officials from the General Directorates of Communicable Disease Control, Surveillance, Infection Control and Prevention and Central Laboratories. The CDC CAT includes 60 indicators assessing RRT capacity and operational function in eight programme areas, which include administrative considerations (14), staffing and rostering (9), training (3), activation and predeployment (11), deployment (8), postdeployment (6), monitoring and evaluation (5) and One Health (4) (online supplemental material). Each indicator is assessed by whether it exists or not, capacity level and operational status. The capacity level is characterised as either (1) *implemented capacity (score of 2*), which indicates full development and institutionalisation of sustainable resources (ie, formalised documents, ensured funding); (2) *partial capacity (score of 1*), which indicates limited competency or proficiency. Resources currently in the development stage and/or established, but sustainability has not been ensured, and (3) *No capacity (score of 0*) indicates lack of development of sustainable resources. Operational status is characterised as either (1) *fully operational (score of 2*), resource is in or ready for use and has demonstrated ability to function as expected/needed, (2) *limited operational functions (score of 1*), resource is restricted in its ability to function as expected/needed and (3) *not operational (score of 0*), resource is not operational (ie, has not been used). If an indicator does not exist, it is given a score of 0 for capacity and operational status.

The baseline assessment was conducted in March 2022 by two of Egypt’s RRT focal points that were nominated by MoHP leadership (*analysis phase*). The two focal points were provided with an orientation of the CAT and how to assess their RRT capacity and operational level. Afterwards, a CDC technical expert confirmed the baseline assessment following indepth discussions on the indicators with the Egypt RRT focal points. As the phased activities were implemented, the 60 indicators were then reassessed by the same CDC technical expert and the two Egypt RRT focal points. During instances of disagreement over how to score indicators, the three reviewers met to discuss differences in scores until an agreement was reached. The phased activities include the following activities: (1) RRT management training and standard operating procedures development workshop (development phase), (2) staffing and training (implementation phase) and (3) monitoring and evaluation frameworks development workshop (development phase).

### Programme development interventions

In July 2022, following the finalisation of a programme development workplan based on baseline assessment results, Egypt MoHP leadership appointed national RRT managers to participate in the RRT Management Training and Standard Operating Procedures (SOPs) Development Workshop (activity 1).^12^ As part of the design and development phase of the RRT programme capacity building, the objective of this activity was to train newly appointed RRT managers on RRT programme management principles and considerations (*design phase*) and develop the first draft of the SOPs of Egypt’s RRT Programme (*development phase*). The SOPs provide step-by-step guidance for the management and operations of Egypt’s RRT during the preparedness and response phase to ensure that they are ready and equipped to respond with a One Health, all-hazards, multisectoral and multidisciplinary approach to all PHEs.

In August 2022, Egypt’s RRT management team began implementation of their RRT programme’s staffing and rostering procedures for identifying and selecting their first cohort of RRT members using agreed on application and interview processes with preidentified inclusion and exclusion criteria (*implementation phase*). 40 RRT members were selected and trained using an adapted version of WHO’s RRT All-Hazards Trainings Package (activity 2; *implementation phase*). The objective of the training and programme orientation is to equip RRT members with the knowledge and tools to deploy, respond and control PHEs and act as a functional multidisciplinary team when requested to deploy.

Lastly, in May 2023, Egypt’s RRT management team conducted a monitoring and evaluation framework development workshop (activity 3; *development phase*). The aim of the workshop was to develop indicators for monitoring and evaluating the programme’s capacity and reach an agreement on variables to collect during the life of the programme.

### Statistical analysis

Statistical analysis was performed using Excel and R Studio.^13 14^ For descriptive statistics, the capacity and operational level scores were represented as a percent score calculated as the current score over the total possible score. The percentage score was calculated at baseline and after each capacity development activity for each of the eight programme areas. Paired t-tests were used to analyse the difference in mean capacity and operational scores between (1) each phase of capacity building and (2) the start and end of the total intervention. This was done both across individual programme areas and across the aggregated indicators. Lastly, the number and percentage of indicators with a point score change of −2 to –1, 0, 1 and 2 between baseline and total intervention was calculated. Indicators with 0-point change were further analysed by score level (0-point, 1-point and 2-point scores) and within programme areas.

### Patient and public involvement

Patients and the public were not involved in any aspect of this research, including study design, data collection, analysis, reporting or dissemination.

## Results

### Capacity level

**Figure 2:**
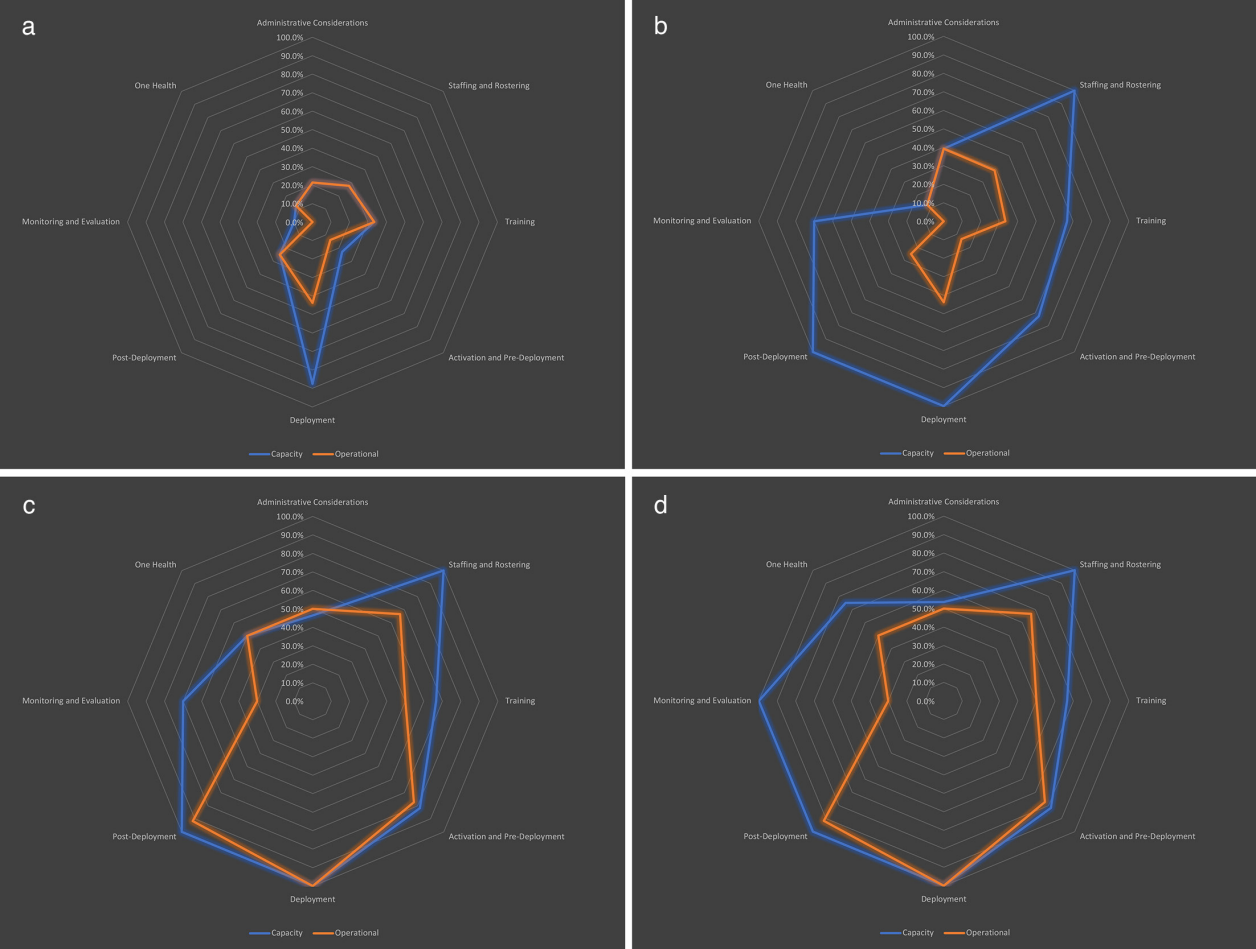
Egypt rapid response team programme’s capacity and operational level scores by programmatic area at baseline (a), post activity 1 (b), post activity 2 (c) and post activity 3 (d).

Figure 2 displays the capacity level scores across the eight programmatic areas as a percentage of the current score over the max score at baseline and post the three interventional activities. At baseline (figure 2a), the scores ranged from 10% (monitoring and evaluation) to 87.5% (deployment) (figure 2b). Capacity scores ranged from 12.5% (One Health) to 100% (staffing and rostering, deployment and postdeployment). Postdeployment (25.0% to 100%) and staffing and rostering (27.8% to 100%) saw the biggest increase after activity 1 (figure 2c). Capacity level scores ranged from 46.4% (administrative considerations) to 100% (deployment, postdeployment, staffing and rostering). One Health showed the greatest improvement between activity 1 and activity 2, improving from 12.5% to 50%. (figure 2d). Capacity level scores ranged from 53.6% (administrative considerations) to 100% (deployment, postdeployment, staffing and rostering, monitoring and evaluation).

Overall, between baseline and the end of the three activities, all programme areas showed statistically significant improvements in capacity scores except three (online supplemental table S1), which were training (0.67; 0.423), deployment (0.25; 0.351) and One Health (1.25; 0.08). Out of all programme areas, monitoring and evaluation (1.80; 0.001) and postdeployment (1.50; 0.007) had the largest mean increases in capacity scores. Between baseline and post activity 1, the overall increase in capacity was 0.77 (0.001). Staffing and rostering (1.22; 0.002), postdeployment (1.50; 0.007), monitoring and evaluation (1.20; 0.004) and activation and predeployment (1.00; 0.008) had the largest average improvements in capacity scores. Improvements in administrative considerations (0.36; 0.054), training (0.67; 0.423), deployment (0.25; 0.351) and One Health (0.00; NA) were not statistically significant. Between activity 1 and activity 2, the overall increase in capacity was 0.12 (0.034). However, there was no significant change in capacity scores in each of the programmatic areas. Between activity 2 and activity 3, the overall increase in capacity was 0.12 (0.007). However, there was no significant change in capacity scores in each of the programmatic areas.

From baseline to the end of the intervention, there were no −2-point and −1-point score changes in capacity level scores across all indicators. There were 2-point score improvements in 25 indicators (41.7%), 1-point score improvements in 12 indicators (20%) and 0-point score net change in 23 indicators (38.3%; table 1) in capacity scores. Over half (55.5%) of the indicators that remained at a score of 0, and all (100%) of the indicators that remained at a score of 1 were in the programme area of administrative considerations.

**Table 1:** Indicators with negative, none or positive change in capacity and operational level scores by post the interventional activities and programmatic areas where indicators remained the same from baseline

			Operational level
		N (%)	N (%)
Indicators with −2-point change		0 (0)	0 (0)
Indicators with −1-point change		0 (0)	0 (0)
Indicators with 0-point change		23 (38.3)	22 (36.6)
	Indicators remained at score of 0	9 (39.1)	15 (68.2)
	Administrative considerations	5 (55.5)	6 (40)
	Staffing and rostering	0 (0)	3 (20)
	Training	1 (11.1)	1 (6.7)
	Activation and predeployment	2 (22.2)	2 (13.3)
	Deployment	0 (0)	0 (0)
	Post Deployment	0 (0)	0 (0)
	Monitoring and evaluation	0 (0)	2 (13.3)
	One Health	1 (11.1)	1 (6.7)
Indicators remained at score of 1		2 (8.7)	3 (13.6)
	Administrative considerations	2 (100)	2 (66.7)
	Staffing and rostering	0 (0)	0 (0)
	Training	0 (0)	0 (0)
	Activation and predeployment	0 (0)	0 (0)
	Deployment	0 (0)	0 (0)
	Post Deployment	0 (0)	0 (0)
	Monitoring and evaluation	0 (0)	0 (0)
	One Health	0 (0)	1 (33.3)
Indicators remained at score of 2		12 (52.2)	4 (18.2)
	Administrative considerations	0 (0)	0 (0)
	Staffing and rostering	1 (8.3)	1 (25)
	Training	1 (8.3)	1 (25)
	Activation and pre-deployment	2 (16.7)	1 (25)
	Deployment	7 (58.3)	00 (0)
	Post Deployment	1 (8.3)	1 (25)
	Monitoring and evaluation	0 (0)	0 (0)
	One Health	0 (0)	0 (0)
Indicators with 1-point change		12 (20.0)	23 (38.3)
Indicators with 2-point change		25 (41.7)	15 (25.0)

### Operational level

Figure 2 displays the operational level scores across the eight programme areas as a percentage of the current score over the max score at baseline and post the three interventional activities. At baseline (figure 2), the scores ranged from 0.00% (monitoring and evaluation) to 43.8% (deployment). No programme area had an operational level score above 50% at baseline. After activity 1 (figure 2), no significant improvement was noted in (monitoring and evaluation) and (deployment) areas. Administrative considerations showed the greatest improvement after activity 1, increasing from 21.4% at baseline to 39.3%. After activity 2 (figure 2), operational level scores ranged from 30% (monitoring and evaluation) to 100% (deployment). Operational level scores improved across all programmatic areas after activity two and were notably highest in postdeployment (25%–91.7%) and deployment (43.8%–100%). After activity 3 (figure 2), no improvement was shown in the operational level scores for both (monitoring and evaluation) and (deployment) areas.

Overall, when analysing the indicators as an aggregate, there was a statistically significant increase in operational level score of 0.88 (p<0.001) between the baseline and the end of the intervention. All programme areas had statistically significant increases in operational level score except monitoring and evaluation (0.60; 0.75) and One Health (0.75; 0.215) (table 1). Of these, postdeployment (1.33; 0.01), activation and predeployment (1.27; .003) and deployment (1.12; 0.001) had the largest improvements in operational level score. Between baseline and activity 1, there was no statistically significant increase in operational level scores overall or by programme area. Between activity 1 and activity 2, the overall increase in operational level was 0.77 (p<0.001). All programme areas had statistically significant increases in operational level score except for three: staffing and rostering (0.56; 0.09), monitoring and evaluation (0.60; 0.07) and One Health (0.75; 0.215). Of these, activation and predeployment (1.27; <0.001), postdeployment (1.33; 0.01) and deployment (1.12; 0.001) had the largest increases. Between activity 2 and activity 3, no statistically significant changes were observed overall (0.00; NA) across each of the programme areas (0.00; NA).

From baseline to the end of the intervention, there were no 2-point or 1-point changes in operational level scores across all indicators. There was a 2-point score improvement in 15 indicators (25.0%), a 1-point score improvement in 23 indicators (38.3%) and a 0-point score change in 22 indicators (36.6%; table 1). Of the indicators that remained at 0-point net change at score of 0, six (40%) were in the administrative section and three (20%) in staffing and rostering. Of the indicators that remained at a 0-point net change at a score of 1, two (66.7%) were in the programme area of administrative consideration.

## Discussion

Since the avian influenza epidemic in 2005, Egypt has responded to a variety of public health emergencies and has conducted RRT trainings to try and enhance their response capacities. As a part of these efforts, in 2022, Egypt adopted the CDC CAT to assess the capacity and operational levels of their current RRT programme. This study aimed to explore Egypt’s RRT Programme’s phased capacity building initiative and the effectiveness of the adopted structured phased approach to significantly enhance the capacity and operational levels of the programme, particularly in the context of PHE preparedness and response. The findings indicate the effectiveness of the phased approach interventions implemented by the Egypt RRT programme, which resulted in significant improvements in both capacity and operational levels across various important programmatic areas.

Overall, the Egypt RRT programme showed a sustained improvement in capacity and operational levels after the three-phased interventions, with over half of the indicators showing a 1-point or 2-point improvement and no decline observed after the phased approach interventions. There was a near-equal distribution of improvement in scores across indicators for both capacity and operational levels. Furthermore, no indicators showed a decline in either operational level or capacity score after the phased intervention. Overall, the greatest impact of the phased approach was obvious in the programme area of postdeployment as it saw the largest improvement in both capacity and operational level scores. Programmatic areas such as monitoring and evaluation and postdeployment had the largest improvements in capacity level scores from baseline. This may be due to a lack of SOPs for several management considerations for these programme areas addressed during the development phase (activity 1). Improvement in operational level was noted in activation, predeployment and postdeployment operations, possibly due to improved capacity levels in those programmatic areas.

The Egypt RRT programme experienced the most improvement in overall capacity level between the analysis phase (baseline) and the development phase (activity 1); whereas overall operational level improved most between the development phase (activity 1) and implementation phase (activity 2). This highlights that specific phases of the phased approach to capacity building target improvements in capacity level or operational level. Most improvements in capacity level were noted post the development phase (activity 1), in programme areas such as staffing and rostering, activation, predeployment, postdeployment and monitoring and evaluation. Activity 1 focuses on developing standard procedures within existing resources to improve the management of these programmatic areas. Programmatic areas that did not have significant improvement in capacity level through the development phase (activity 1) were training, deployment and One Health. This may be due to previously existing capacities in Egypt at baseline (ie, training and deployment), with minor improvements during the development phase (activity 1) or because the development phase activity does not effectively address indicators within those programmatic areas (ie, administrative considerations and One Health).

Most improvement in the operational level was noted post implementation phase (activity 2) in programmatic areas such as administrative considerations, training, activation and predeployment, deployment and postdeployment. Improvements in the programme area of training coincided with the RRT All-Hazards Training conducted for the first cohort of the RRT programme.^15^ However, at the end of the training, Egypt RRT managers had to activate the programme to investigate an outbreak in a remote village, which immediately operationalised programmatic areas of administrative considerations, activation and predeployment, deployment and postdeployment procedures. The team successfully conducted epidemiological investigation, contact tracing, implemented preventive and control measures and performed risk communication and community engagement, demonstrating the practical effectiveness of the capacity building interventions. Additionally, Egypt has since responded to several other public health emergencies, including foodborne illness outbreaks in three governorates and suspected viral haemorrhagic fever cases. Furthermore, RRT participated in multiple mass gathering events in Egypt such as United Nations Climate Change Conference (COP27). The RRT developed health emergency contingency plans and participated in a top table exercise for health emergency in the preparedness phase. While teams were deployed during the conference for outbreak investigation and response system in an operational process for health emergencies. These subsequent deployments have consistently shown reduced response times, improved data collection and reporting and enhanced coordination between national and local health authorities, providing real-world validation of the training programme and immediate operationalisation of the newly developed SOPs.

Programmatic areas that did not have significant improvement at the operational level through the phased approach were monitoring and evaluation and One Health. This is likely due to capacities within monitoring and evaluation, and One Health has not been developed or fully developed to begin implementation.

Indicators that did not improve from the baseline score of 0 or one in both capacity and operational levels were from the programmatic area of administrative considerations. Further review of those indicators noted that they cover areas such as the establishment of policy related to a multisectoral approach to emergency response and budget development and sustainable government funding for core programme activities and resources related to management, deployment equipment and training. Development of a workplan for programme development was conducted during the design phase of the phased approach. However, these results indicate that further efforts in line with multisectoral policy development and sustainable budgetary considerations could have been conducted during the design phase to facilitate leadership support and sustainability of capacity and operational level improvements of the programme. This study had some limitations. First, the end data were collected relatively soon after the last activity, which may impact the level at which this paper can assess the long-term sustainability of the interventions. Second, the data were collected in the setting of a single country, which limits the total sample size available for analysis. The single country setting also makes the results harder to extrapolate to other contexts. Third, data collection was conducted by individuals closely related to the development of the programme and may have benefited from having external objective evaluators. Lastly, Egypt did not conduct evaluation phase activities for their RRT programme and, therefore, could not determine the accountability of programme processes. In future studies, working with aggregated data from multiple countries may help address these limitations.

Continuous assessment of the Egypt RRT programme allowed tracking of programme capacity and operational improvement and sustainability over time. Since completing the programme development interventions for the RRT programme, Egypt has formalised its One Health Strategic Plan.^16^ As a result, a One Health Department within the Egypt MoHP was established, where the RRT programme now resides, to coordinate a multisectoral approach to responding to PHEs with sustained annual government funding. This paramount step taken at a policy and administrative level paves the way for the Egypt RRT Managers to begin addressing the current resource gaps to further develop and operationalise the programme areas already developed (ie, monitoring and evaluation framework) with minimal reliance on external partner support.

Similar initiatives demonstrate the unique effectiveness of Egypt’s systematic approach. A study in the Eastern Mediterranean Region (EMR) including (Egypt, Jordan, Morocco, Sudan, Tunisia) implemented RRT capacity building through traditional training workshops and achieved modest improvements in response times but struggled with sustainability and institutional integration.^17 18^ Another study reported systemic gaps in responding to public health events effectively in the EMR such as institutionalisation of rapid response workforce, limited operational capacity and maintaining trained personnel rosters.^19^ Egypt’s ADDIE-based approach distinguished itself by achieving both individual and institutional improvements simultaneously. The phased approach’s emphasis on developing SOPs and monitoring frameworks appears to address common sustainability challenges identified in similar programmes across low- and middle-income countries.

In conclusion, lessons learnt from Egypt’s experience to systematically verify the ADDIE model phased approach highlight that it could be a potential model for future emergency capacity building initiatives in place of an individual approach to training. In addition, this experience suggests that future use of the phased approach to capacity building might be improved by pre-established multisectoral policy development and sustainable budgetary considerations prior to or during the design phase. This study is a primary building block to assist in the facilitation of official endorsement and leadership support. Implementing these fundamental elements during the design phase may be a key driver to programme establishment and its sustainability over time.

## Supplementary material

10.1136/bmjgh-2025-019008 online supplemental file 1

## Data Availability

Data are available upon reasonable request.

## References

[1] World Health Organization (2005). International health regulations. https://iris.who.int/handle/10665/246107.

[2] World Health Organization (2022). Joint external evaluation tool: international health regulations (2005). https://iris.who.int/handle/10665/357087.

[3] Nguyen L, Brown SM, Couture A (2021). Global Health Security Preparedness and Response: An Analysis of the Relationship between Joint External Evaluation Scores and COVID-19 Response Performance. BMJ Open.

[4] Kurt DS (2017). ADDIE model: instructional design. https://educationaltechnology.net/the-addie-model-instructional-design/.

[5] Noh J, Oh EG, Kim SS (2020). Development and evaluation of a multimodality simulation disaster education and training program for hospital nurses. Int J of Nursing Practice.

[6] Greiner A, Stehling-Ariza T, Hoffman A (2020). Guidance for U.S. Centers for Disease Control and Prevention staff for the establishment and management of public health rapid response teams for disease outbreaks.

[7] Preventive Medicine M (2016). The ministry of health and population’s plan to combat avian influenza. https://www.mohp.gov.eg/UserFiles/LibraryFiles/605869.pdf?csrt=10425223230628563766.

[8] Shamseldein S, Azqul M, Samy S (2020). A large-scale outbreak of botulism associated with a traditional celebratory Egyptian fish dish in five governorates - Lower Egypt, 2019: a teaching case-study. Pan Afr Med J.

[9] Hassan H, Abo ElSood H, Abd ElGawad B (2022). The value of contact tracing and isolation in mitigation of COVID-19 epidemic: findings from outbreak investigation of COVID-19 onboard Nile Cruise Ship, Egypt, March 2020. BMJ Glob Health.

[10] National Center for Immunization and Respiratory Diseases (U.S.). Division of Viral Diseases (2021). Emergency Preparedness and Response Capacity Assessment Tool.

[11] publisher E (2024). Building rapid response capacities for a timely and effective national response to disease outbreaks in Egypt. http://www.emro.who.int/pandemic-epidemic-diseases/news/building-rapid-response-capacities-for-a-timely-and-effective-national-response-to-disease-outbreaks-in-egypt.html.

[12] (2024). Workshop to review and update the national rapid response team standard operating procedures in Egypt. https://emphnet.net/en/resources/news/2022/workshop-to-review-and-update-the-national-rapid-response-team-standard-operating-procedures-in-egypt/.

[13] Microsoft Corporation Microsoft excel. microsoft excel for microsoft 365 MSO (version 2302 build 16.0.16130.20586) 64-bit. https://office.microsoft.com/excel.

[14] RStudio Team (2020). RStudio: Integrated Development for R.

[15] (2024). Block 4 - RRT advanced training package | HSLP. https://extranet.who.int/hslp/content/all-hazard-rrt-training-package-version-20.

[16] (2024). Egypt launches one health approach to safeguard human, animal, and environmental health - health - Egypt. https://english.ahram.org.eg/NewsContent/1/1236/495496/Egypt/Health/Egypt-launches-One-Health-Approach-to-safeguard-hu.aspx.

[17] Araj R, Odatallah A, Mofleh J (2019). Rapid Response Teams’ Initiative: Critical Role and Impact on National and Eastern Mediterranean Regional Emergency Management Capacity Building. JMIR Public Health Surveill.

[18] Elnosserry S, Buliva E, Abdalla Elkholy A (2024). Rapid response teams in the Eastern Mediterranean Region: Results from the baseline survey of country-level capacities, operations and outbreak response capabilities. Glob Public Health.

[19] Khader Y, Shalabi D, Daoud L (2025). The role of public health networks in strengthening public health systems: the case of EMPHNET in the Eastern Mediterranean region. Front Public Health.

