# Supplementary material for: A novel phased approach to rapid response capacity strengthening in public health emergencies: Egypt’s experience
Source: BMJ Glob Health. 2026 Jan 8;11(1):e019008. doi: 10.1136/bmjgh-2025-019008 (PMC13059850; doi:10.1136/bmjgh-2025-019008)
Supplement: online supplemental file 1 [file bmjgh-11-1-s001.docx]

**A novel phased approach to rapid response capacity strengthening in public health emergencies: Egypt’s experience**

**Supplementary Materials**

Table S1: Mean difference in capacity and operational level scores from baseline and between activities 1, 2, and 3.

|  | **Administrative considerations** | | **Staffing and rostering** | | **Training** | | **Activation and Pre-deployment** | | **Deployment** | | **Post-deployment** | | **Monitoring and evaluation** | | **One Health** | | **Total** | |
| --- | --- | --- | --- | --- | --- | --- | --- | --- | --- | --- | --- | --- | --- | --- | --- | --- | --- | --- |
|  | Δ mean (SD) | p-value | Δ mean (SD) | p-value | Δ mean (SD) | p-value | Δ mean (SD) | p-value | Δ mean (SD) | p-value | Δ mean (SD) | p-value | Δ mean (SD) | p-value | Δ mean (SD) | p-value | Δ mean (SD) | p-value |
| **Capacity level** | | | | | | | | | | | | | | | | | | |
| **Baseline and Activity 1** | 0.36 (0.63) | 0.054 | 1.22 (0.83) | 0.002 | 0.67 (1.15) | 0.423 | 1.00 (1.00) | 0.008 | 0.25 (0.71) | 0.351 | 1.50 (0.84) | 0.007 | 1.20 (0.45) | 0.004 | 0.00 (0.00) | NA | 0.77 (0.87) | < 0.001 |
| **Activity 1 and Activity 2** | 0.14 (0.36) | 0.164 | 0.00 (0.00) | NA | 0.00 (0.00) | NA | 0.18 (0.60) | 0.34 | 0.00 (0.00) | NA | 0.00 (0.00) | NA | 0.00 (0.00) | NA | 0.75 (0.96) | 0.214 | 0.12 (0.42) | 0.034 |
| **Activity 2 and Activity 3** | 0.14 (0.36) | 0.165 | 0.00 (0.00) | NA | 0.00 (0.00) | NA | 0.00 (0.00) | NA | 0.00 (0.00) | NA | 0.00 (0.00) | NA | 0.60 (0.55) | 0.07 | 0.50 (0.58) | 0.181 | 0.12 (0.32) | 0.007 |
| **Baseline and Activity 3** | 0.64 (0.74) | 0.007 | 1.22 (0.83) | 0.002 | 0.67 (1.15) | 0.423 | 1.18 (0.98) | 0.003 | 0.25 (0.71) | 0.351 | 1.50 (0.84) | 0.007 | 1.80 (0.45) | 0.001 | 1.25 (0.96) | 0.08 | 1.00 (0.90) | < 0.001 |
| **Operational level** | | | | | | | | | | | | | | | | | | |
| **Baseline and Activity 1** | 0.36 (0.63) | 0.055 | 0.22 (0.44) | 0.17 | 0.00 (0.00) | NA | 0.00 (0.00) | NA | 0.00 (0.00) | NA | 0.00 (0.00) | NA | 0.00 (0.00) | NA | 0.00 (0.00) | NA | 0.12 (0.37) | 0.085 |
| **Activity 1 and Activity 2** | 0.21 (0.43) | < 0.001 | 0.56 (0.88) | 0.09 | 0.33 (0.58) | < 0.001 | 1.27 (0.90) | < 0.001 | 1.12 (0.35) | 0.001 | 1.33 (0.82) | 0.01 | 0.60 (0.55) | 0.07 | 0.75 (0.96) | 0.215 | 0.77 (0.79) | < 0.001 |
| **Activity 2 and Activity 3** | 0.00 (0.00) | NA | 0.00 (0.00) | NA | 0.00 (0.00) | NA | 0.00 (0.00) | NA | 0.00 (0.00) | NA | 0.00 (0.00) | NA | 0.00 (0.00) | NA | 0.00 (0.00) | NA | 0.00 (0.00) | NA |
| **Baseline and Activity 3** | 0.57 (0.76) | 0.014 | 0.78 (0.83) | 0.02 | 0.33 (0.58) | < 0.001 | 1.27 (0.90) | < 0.001 | 1.12 (0.35) | 0.001 | 1.33 (0.82) | 0.01 | 0.60 (0.55) | 0.07 | 0.75 (0.96) | 0.215 | 0.88 (0.78) | < 0.001 |

**EmergencyPreparedness and Response Capacity Assessment Tool (CAT) - Version 2**

| Date Completed: |
| --- |
| Country: |

| **Representative(s) Completing Form: (Name, agency, role, e-mail)** | | | | | | | | | |
| --- | --- | --- | --- | --- | --- | --- | --- | --- | --- |
| **Country Level of Focus:**  **(National/central level, subnational/provincial level (please specify), etc.)** | | | | | | | | | |
| **Agency/ Organization(s):** | | | | | | | | | |
| # | Public Health Rapid Response Teams (RRTs) | Exists | Date Implemented/ Last Updated | Operational Status | Lead Agency | Capacity | Priority | In-Country Point of Contact (Name, Agency, Role, Contact Information) | Comments/  Additional Information |
| # | **Administrative Considerations** | **Exists** | **Date Implemented/ Last Updated** | **Operational Status** | **Lead Agency** | **Capacity** | **Priority** | **In-Country Point of Contact (Name, Agency, Role, Contact Information)** | **Comments/ Additional Information** |
| 1 | RRT SOPs drafted and finalized by all relevant stakeholders and members of leadership mentioned in the documents | Choose an item. | Click or tap to enter a date. | Choose an item. |  | Choose an item. | Choose an item. |  |  |
| 2 | RRT or similar multidisciplinary surge program with members trained, organized, and ready to deploy exists at the national level | Choose an item. | Click or tap to enter a date. | Choose an item. |  | Choose an item. | Choose an item. |  |  |
| 3 | Identified human (individual vs team) and technological resources (laptops, internet, survey tools, dashboards, etc.) to manage/support the RRT program in peacetime | Choose an item. | Click or tap to enter a date. | Choose an item. |  | Choose an item. | Choose an item. |  |  |
| 4 | Identified human (individual vs team) and technological resources (laptops, internet, survey tools, dashboards, etc.) to manage/support the RRT program during response | Choose an item. | Click or tap to enter a date. | Choose an item. |  | Choose an item. | Choose an item. |  |  |
| 5 | RRT management staff is equipped with the necessary technological tools to collect and manage data and information pertaining to the RRT members (i.e., laptops, internet,survey tools, dashboards, etc.) | Choose an item. | Click or tap to enter a date. | Choose an item. |  | Choose an item. | Choose an item. |  |  |
| 6 | RRT management staff have been trained on how to collect and manage data and information pertaining to the RRT members | Choose an item. | Click or tap to enter a date. | Choose an item. |  | Choose an item. | Choose an item. |  |  |
| 7 | Established legal contracts/agreements for multisectoral RRT mobilization within the RRT administrative agency | Choose an item. | Click or tap to enter a date. | Choose an item. |  | Choose an item. | Choose an item. |  |  |
| 8 | Established legal contracts/agreements with other national ministries/agencies for multisectoral RRT mobilization | Choose an item. | Click or tap to enter a date. | Choose an item. |  | Choose an item. | Choose an item. |  |  |
| 9 | Established RRT budget considering the requisite preparedness and response operations (i.e., salary, per diem, training programs, travel, equipment, vaccinations, database maintenance, etc.) | Choose an item. | Click or tap to enter a date. | Choose an item. |  | Choose an item. | Choose an item. |  |  |
| 10 | Established sustainable financial support for the RRT program from national ministries/agencies to support RRT administrative costs, member readiness, and deployments | Choose an item. | Click or tap to enter a date. | Choose an item. |  | Choose an item. | Choose an item. |  |  |
| 11 | Established sustainable financial support for the RRT program from external agencies/organizations to support RRT administrative costs, member readiness, and deployment | Choose an item. | Click or tap to enter a date. | Choose an item. |  | Choose an item. | Choose an item. |  |  |
| 12 | Established functioning resource coordination and mobilization mechanisms (i.e., EOC equipment, travel logistics, field transportation, etc.) | Choose an item. | Click or tap to enter a date. | Choose an item. |  | Choose an item. | Choose an item. |  |  |
| 13 | RRT safety and security processes have been outlined and shared with RRT members | Choose an item. | Click or tap to enter a date. | Choose an item. |  | Choose an item. | Choose an item. |  |  |
| 14 | Established system for identifying and procuring necessary RRT equipment | Choose an item. | Click or tap to enter a date. | Choose an item. |  | Choose an item. | Choose an item. |  |  |
| # | **Staffing and Rostering** | **Exists** | **Date Implemented/ Last Updated** | **Operational Status** | **Lead Agency** | **Capacity** | **Priority** | **In-Country Point of Contact (Name, Agency, Role, Contact Information)** | **Comments/ Additional Information** |
| 1 | Fully dedicated RRT management team with staff members integrated into the country’s emergency response system | Choose an item. | Click or tap to enter a date. | Choose an item. |  | Choose an item. | Choose an item. |  |  |
| 2 | RRT management personnel include the following responsibilities: deployment coordination, data manager, training coordinator, monitoring, and evaluation | Choose an item. | Click or tap to enter a date. | Choose an item. |  | Choose an item. | Choose an item. |  |  |
| 3 | Established RRT recruitment and onboarding standard operating procedures (e.g., candidate inclusion/exclusion criteria, multisectoral/multidisciplinary candidate sources, database platform/variables, data collection, roster updates, mitigating roster attrition, etc.) | Choose an item. | Click or tap to enter a date. | Choose an item. |  | Choose an item. | Choose an item. |  |  |
| 4 | Recruitment process targets multisectoral RRT members with differing technical expertise to best utilize a One Health approach to outbreak response | Choose an item. | Click or tap to enter a date. | Choose an item. |  | Choose an item. | Choose an item. |  |  |
| 5 | Formalized recruitment process with inclusion/exclusion criteria of selecting highly skilled and experienced individuals for the RRT roster | Choose an item. | Click or tap to enter a date. | Choose an item. |  | Choose an item. | Choose an item. |  |  |
| 6 | Annual recruitment of new RRT members | Choose an item. | Click or tap to enter a date. | Choose an item. |  | Choose an item. | Choose an item. |  |  |
| 7 | Each RRT member agrees to commit to the RRT program for the duration of the term specified by the governing public health agency | Choose an item. | Click or tap to enter a date. | Choose an item. |  | Choose an item. | Choose an item. |  |  |
| 8 | Each RRT member has obtained the necessary approvals as dictated by the relevant SOPs for participation in the rapid response team program | Choose an item. | Click or tap to enter a date. | Choose an item. |  | Choose an item. | Choose an item. |  |  |
| 9 | Identified and trained multidisciplinary rapid response workforce/surge pool | Choose an item. | Click or tap to enter a date. | Choose an item. |  | Choose an item. | Choose an item. |  |  |
| # | **Training** | **Exists** | **Date Implemented/ Last Updated** | **Operational Status** | **Lead Agency** | **Capacity** | **Priority** | **In-Country Point of Contact (Name, Agency, Role, Contact Information)** | **Comments/ Additional Information** |
| 1 | Established onboarding training for new RRT members including the provision of initial all-hazards technical, operational, and administrative training | Choose an item. | Click or tap to enter a date. | Choose an item. |  | Choose an item. | Choose an item. |  |  |
| 2 | Established continuing education sessions on supplementary technical skills related to field deployments, soft skills, and response updates, to be completed on annual basis | Choose an item. | Click or tap to enter a date. | Choose an item. |  | Choose an item. | Choose an item. |  |  |
| 3 | Developed internal repository of disease or event specific just-in-time trainings | Choose an item. | Click or tap to enter a date. | Choose an item. |  | Choose an item. | Choose an item. |  |  |
| # | **Activation and Pre-Deployment** | **Exists** | **Date Implemented/ Last Updated** | **Operational Status** | **Lead Agency** | **Capacity** | **Priority** | **In-Country Point of Contact (Name, Agency, Role, Contact Information)** | **Comments/ Additional Information** |
| 1 | Developed pre-deployment standard operating procedures (SOPs) (e.g., briefing, just-in-time training, equipment, etc.) | Choose an item. | Click or tap to enter a date. | Choose an item. |  | Choose an item. | Choose an item. |  |  |
| 2 | Established defined, written criteria for activation of RRT program to deploy RRT members to the field | Choose an item. | Click or tap to enter a date. | Choose an item. |  | Choose an item. | Choose an item. |  |  |
| 3 | RRT pre-deployment briefing process and information outlined | Choose an item. | Click or tap to enter a date. | Choose an item. |  | Choose an item. | Choose an item. |  |  |
| 4 | Pre-deployment requirements identified, communicated, documented for every newly onboarded RRT member | Choose an item. | Click or tap to enter a date. | Choose an item. |  | Choose an item. | Choose an item. |  |  |
| 5 | Developed safety and security evacuation plans (including protection and evacuation plans) prior to deployment with national and field response team | Choose an item. | Click or tap to enter a date. | Choose an item. |  | Choose an item. | Choose an item. |  |  |
| 6 | Identified resources and mechanisms to ensure RRT safety, health, and wellbeing including in RRT planning (e.g., medical/disability/life insurance, medical care, mental health care, emergency evacuation etc.) | Choose an item. | Click or tap to enter a date. | Choose an item. |  | Choose an item. | Choose an item. |  |  |
| 7 | Identified resources and mechanisms to ensure RRT safety, health, and wellbeing are included in response plans | Choose an item. | Click or tap to enter a date. | Choose an item. |  | Choose an item. | Choose an item. |  |  |
| 8 | Outlined the requirement for healthcare insurance by the RRT member’s respective national ministries/agency/external partner | Choose an item. | Click or tap to enter a date. | Choose an item. |  | Choose an item. | Choose an item. |  |  |
| 9 | Outlined access to mental health care by a specialist pre-, during and post- deployment for RRT members | Choose an item. | Click or tap to enter a date. | Choose an item. |  | Choose an item. | Choose an item. |  |  |
| 10 | Described how RRT members can access necessary RRT equipment and supplies, personal protective equipment, and mandatory vaccinations prior to deployment | Choose an item. | Click or tap to enter a date. | Choose an item. |  | Choose an item. | Choose an item. |  |  |
| 11 | RRT pre-deployment just-in-time training identified and conducted | Choose an item. | Click or tap to enter a date. | Choose an item. |  | Choose an item. | Choose an item. |  |  |
| # | **Deployment** | **Exists** | **Date Implemented/ Last Updated** | **Operational Status** | **Lead Agency** | **Capacity** | **Priority** | **In-Country Point of Contact (Name, Agency, Role, Contact Information)** | **Comments/ Additional Information** |
| 1 | Developed deployment SOPs (e.g., communication, reporting, team evolution, etc.) | Choose an item. | Click or tap to enter a date. | Choose an item. |  | Choose an item. | Choose an item. |  |  |
| 2 | Developed standard terms of reference for commonly deployed positions | Choose an item. | Click or tap to enter a date. | Choose an item. |  | Choose an item. | Choose an item. |  |  |
| 3 | Developed field information sharing protocols and procedures | Choose an item. | Click or tap to enter a date. | Choose an item. |  | Choose an item. | Choose an item. |  |  |
| 4 | Developed standard RRT reporting measures including reporting requirements and supporting templates | Choose an item. | Click or tap to enter a date. | Choose an item. |  | Choose an item. | Choose an item. |  |  |
| 5 | Defined criteria for demobilization of RRT member(s) to return home from the field | Choose an item. | Click or tap to enter a date. | Choose an item. |  | Choose an item. | Choose an item. |  |  |
| 6 | Developed demobilization procedures (e.g., notification, close-out procedures) | Choose an item. | Click or tap to enter a date. | Choose an item. |  | Choose an item. | Choose an item. |  |  |
| 7 | Identified handoff process and transfer of information from outgoing to incoming RRT member to the field Defined mechanisms for RRT member coordination and reporting to the RRT manager or Emergency Operating Center (EOC) | Choose an item. | Click or tap to enter a date. | Choose an item. |  | Choose an item. | Choose an item. |  |  |
| 8 | Defined mechanisms for RRT member coordination and reporting to the RRT manager or Emergency Operating Center (EOC) | Choose an item. | Click or tap to enter a date. | Choose an item. |  | Choose an item. | Choose an item. |  |  |
| # | **Post-deployment** | **Exists** | **Date Implemented/ Last Updated** | **Operational Status** | **Lead Agency** | **Capacity** | **Priority** | **In-Country Point of Contact (Name, Agency, Role, Contact Information)** | **Comments/ Additional Information** |
| 1 | Developed post-deployment SOPs (e.g., demobilization criteria, debriefs, etc.) | Choose an item. | Click or tap to enter a date. | Choose an item. |  | Choose an item. | Choose an item. |  |  |
| 2 | Developed process for providing medical and/or mental health resources after deployment | Choose an item. | Click or tap to enter a date. | Choose an item. |  | Choose an item. | Choose an item. |  |  |
| 3 | Developed process for administrative support to returning RRT members (i.e., procurement of travel expenses, per diem, time off, etc.) | Choose an item. | Click or tap to enter a date. | Choose an item. |  | Choose an item. | Choose an item. |  |  |
| 4 | Described the debrief and/or after-action review process | Choose an item. | Click or tap to enter a date. | Choose an item. |  | Choose an item. | Choose an item. |  |  |
| 5 | Described the process of handling sensitive information | Choose an item. | Click or tap to enter a date. | Choose an item. |  | Choose an item. | Choose an item. |  |  |
| 6 | Identified information to be collected during the debrief and/or after-action review | Choose an item. | Click or tap to enter a date. | Choose an item. |  | Choose an item. | Choose an item. |  |  |
| # | **Monitoring and Evaluation** | **Exists** | **Date Implemented/ Last Updated** | **Operational Status** | **Lead Agency** | **Capacity** | **Priority** | **In-Country Point of Contact (Name, Agency, Role, Contact Information)** | **Comments/ Additional Information** |
| 1 | Developed monitoring, evaluation and improvement planning of the RRT program | Choose an item. | Click or tap to enter a date. | Choose an item. |  | Choose an item. | Choose an item. |  |  |
| 2 | Established system for data collection and data management of RRT activities conducted | Choose an item. | Click or tap to enter a date. | Choose an item. |  | Choose an item. | Choose an item. |  |  |
| 3 | Developed evaluation tools to measure RRT activities in the field (e.g. Surveys, focus groups, interviews, observation etc.) | Choose an item. | Click or tap to enter a date. | Choose an item. |  | Choose an item. | Choose an item. |  |  |
| 4 | Developed tracking process and timeline for modifying guidelines, SOPs, process, and trainings | Choose an item. | Click or tap to enter a date. | Choose an item. |  | Choose an item. | Choose an item. |  |  |
| 5 | RRT Monitoring and Evaluation has corrective action planning leading to administrative/training changes in the RRT program | Choose an item. | Click or tap to enter a date. | Choose an item. |  | Choose an item. | Choose an item. |  |  |
| # | **One Health** | **Exists** | **Date Implemented/ Last Updated** | **Operational Status** | **Lead Agency** | **Capacity** | **Priority** | **In-Country Point of Contact (Name, Agency, Role, Contact Information)** | **Comments/ Additional Information** |
| 1 | One Health has been incorporated into existing emergency response infrastructure | Choose an item. | Click or tap to enter a date. | Choose an item. |  | Choose an item. | Choose an item. |  |  |
| 2 | Established mechanisms in place to respond to zoonotic diseases and other | Choose an item. | Click or tap to enter a date. | Choose an item. |  | Choose an item. | Choose an item. |  |  |
| 3 | One Health integrated into training requirements for RRT members | Choose an item. | Click or tap to enter a date. | Choose an item. |  | Choose an item. | Choose an item. |  |  |
| 4 | One Health professionals from each sector trained and ready to effectively respond to outbreaks and public health emergencies | Choose an item. | Click or tap to enter a date. | Choose an item. |  | Choose an item. | Choose an item. |  |  |
